# Pediatric Integrative Medicine in Residency Program: Relationship between Lifestyle Behaviors and Burnout and Wellbeing Measures in First-Year Residents

**DOI:** 10.3390/children5040054

**Published:** 2018-04-23

**Authors:** Hilary McClafferty, Audrey J. Brooks, Mei-Kuang Chen, Michelle Brenner, Melanie Brown, Anna Esparham, Dana Gerstbacher, Brenda Golianu, John Mark, Joy Weydert, Ann Ming Yeh, Victoria Maizes

**Affiliations:** 1 Department of Medicine, Arizona Center for Integrative Medicine, University of Arizona, Tucson, AZ 85724, USA; kuang@email.arizona.edu (M.-K.C.); vmaizes@email.arizona.edu (V.M.); 2 Department of Pediatrics, Eastern Virginia Medical School/Children’s Hospital of the King’s Daughters, Norfolk, VA 23507, USA; michelle.brenner@chkd.org; 3 Department of Pediatrics, University of Chicago Comer Children’s Hospital, Chicago, IL 60637, USA; Melanie.Brown@childrensmn.org; 4 Department of Pediatrics, University of Kansas School of Medicine, Kansas City, KS 66160, USA; aeesparham@cmh.edu (A.E.); jweydert@kumc.edu (J.W.); 5 Department of Pediatrics, Stanford University School of Medicine, Palo Alto, CA 94304, USA; gerst1@stanford.edu (D.G.); jmark@stanford.edu (J.M.); annming@stanford.edu (A.M.Y.); 6 Department of Anesthesiology and Pain Management, Stanford University School of Medicine, Palo Alto, CA 94304, USA; bgolianu@stanford.edu

**Keywords:** burnout, pediatrics, residents, preventive lifestyle behaviors, resilience

## Abstract

It is widely recognized that burnout is prevalent in medical culture and begins early in training. Studies show pediatricians and pediatric trainees experience burnout rates comparable to other specialties. Newly developed Accreditation Council for Graduate Medical Education (ACGME) core competencies in professionalism and personal development recognize the unacceptably high resident burnout rates and present an important opportunity for programs to improve residents experience throughout training. These competencies encourage healthy lifestyle practices and cultivation of self-awareness, self-regulation, empathy, mindfulness, and compassion—a paradigm shift from traditional medical training underpinned by a culture of unrealistic endurance and self-sacrifice. To date, few successful and sustainable programs in resident burnout prevention and wellness promotion have been described. The University of Arizona Center for Integrative Medicine Pediatric Integrative Medicine in Residency (PIMR) curriculum, developed in 2011, was designed in part to help pediatric programs meet new resident wellbeing requirements. The purpose of this paper is to detail levels of lifestyle behaviors, burnout, and wellbeing for the PIMR program’s first-year residents (*N* = 203), and to examine the impact of lifestyle behaviors on burnout and wellbeing. The potential of the PIMR to provide interventions addressing gaps in lifestyle behaviors with recognized association to burnout is discussed.

## 1. Introduction

Medical school and residency training are known for their scientific rigor and daunting hours of service [1,2]. Compounding burnout experienced in medical school, the life of a resident is dominated by exposure to significant human suffering, high levels of responsibility, lack of day-to-day control, steep learning curves, limited regeneration time, and chronic sleep deprivation. These realities, in conjunction with patient and family demands, subordinate ranking in the medical hierarchy, and other stressors predispose residents to high burnout prevalence [1,2].

Studies show that pediatricians and pediatric trainees experience burnout at rates comparable to other specialties, with higher prevalence in some high acuity pediatric subspecialties such as neonatology, intensive care, and hematology-oncology [3,4,5,6]. Across medical specialties and training levels, burnout has been associated with an increase in medical errors, lower adherence to best practices, substance abuse, self-medication, and poorer patient outcomes (for example, longer hospital stay), in addition to increased rates of clinician’s depression, suicidal ideation, and completed suicide [7]. It has been widely reported that effective approaches must include changes at organizational, institutional, and individual levels to address the outdated culture of unrealistic endurance still pervasive in medicine [8].

Newly developed Accreditation Council for Graduate Medical Education (ACGME) core competencies in professionalism and personal development recognize the unacceptably high burnout rates in residents and present an important opportunity for programs to improve the experience of residents at all stages of training [9].

In addition to encouraging healthy lifestyle practices, the core competencies encourage cultivation of self-awareness, self-regulation skills, empathy, mindfulness, and compassion. They also highlight the need for innovation in clinical training and generate questions about how to best teach and measure success in these new areas. This endeavor provides important opportunities to introduce proactive approaches to burnout through a multidimensional model and development of educational curricula that emphasize and measure wellbeing at all phases of medical training.

To date, along with development of the new core competencies, purposeful steps taken by the ACGME include national symposia led by expert national faculty to identify actionable issues, new emphasis on education on physician wellbeing, and recommendations for annual measurement of resident burnout through its Clinical Learning Environmental Review (CLER) program which evaluates the quality of the program learning environment [10].

Despite these significant initiatives, few successful, sustainable programs in resident burnout prevention and wellness promotion have been described. Pediatricians are uniquely positioned to develop national initiatives for solution-based approaches to burnout, in part due to the timely initiation of the 2010 Pediatric Milestone Project, a collaboration between the ACGME and the American Board of Pediatrics (ABP) dedicated to development of revised core competencies. The University of Arizona Center for Integrative Medicine Pediatric Integrative Medicine in Residency (PIMR) curriculum was developed in 2011 and refined in 2012 to include a robust Self-Care unit, partly in response to the new priorities in the revised national pediatric training competencies targeting resident wellbeing outlined by the ACGME.

The 100-h curriculum is an interactive hybrid online-onsite program and has been described in detail in an earlier publication [11]. The program has been piloted since 2012 at the University of Arizona, Stanford University, the University of Kansas, the University of Chicago, and Eastern Virginia Medical School/Children’s Hospital of the King’s Daughters. The topics covered include: Self-; nutrition and physical activity; mind-body therapies; dietary supplements; whole systems of medicine; and clinical applications.

The curriculum was developed with the two-fold purpose of embedding foundational education in pediatric integrative medicine into residency training and introducing a ‘train the trainer’ model in teaching healthy lifestyle habits. In anticipation of high levels of burnout in incoming residents, the Self-Care unit was designed to provide an evidence-based ‘blueprint’ for learning about factors associated with lower burnout prevalence in medical trainees including: physical activity, sleep, stress management, mindfulness, and nutrition, thereby increasing resident knowledge about self-care and also providing a useful educational tool for residency programs to address new ACGME resident wellness competency requirements. An integrated evaluation arm measures resident burnout and lifestyle behaviors, and gathers feedback on perceived quality and relevance of the curriculum at four points (beginning, end Post Graduate Year (PGY) 1, end PGY2, end PGY3) of residency training.

We readily acknowledge that change is urgently needed on multiple levels to address the serious issues of preventing burnout and promoting wellbeing in medical trainees, ideally starting in pre-medical and medical students. We believe our study findings detailing the incoming resident levels of burnout, wellbeing, and lifestyle behaviors and examining the relationship between resident lifestyle habits and burnout and wellbeing measures reinforce this point and begin to explore areas of potential progress. The overarching purpose of this paper is to document the concerning state of burnout in early pediatric trainees and to examine the potential of the PIMR curriculum to provide interventions that address gaps in lifestyle behaviors with recognized association to burnout, and how they might be introduced into residency training.

## 2. Methods

Approval for the study was granted by the University of Arizona Institutional Review Board (Approval #12049200). First-year pediatric residents from five residencies participating in the PIMR program completed standardized wellbeing measures at the start of residency. The PIMR sites include academic and community-based programs led by pediatric faculty with fellowship training in integrative medicine, a field that blends conventional and evidence-based complementary medicine and prioritizes preventive health. Information about existing onsite physician wellness activities was collected and is presented. Data is being collected as part of a longitudinal study on resident wellbeing, burnout and lifestyle behaviors and the longitudinal impact of the PIMR curriculum.

### 2.1. Measures

Residents completed eight widely used, established scales assessing dimensions of well-being: perceived stress, depression symptoms, burnout (emotional exhaustion, depersonalization), life satisfaction, affect, mindfulness, emotional intelligence, and physician empathy (Table 1). A measure of lifestyle behaviors, the Arizona Lifestyle Inventory (ALI) [12], was also administered. The ALI was developed to assess changes in lifestyle behaviors in residents. The domains and items were identified from literature on evidence-based preventive services as well as other areas emphasized by integrative medicine practitioners. The items were reviewed by an expert panel of integrative medicine physicians and were revised based on their comments. The ALI primarily assesses the frequency of behaviors in the past seven days in the areas of diet/nutrition, exercise, mind-body/spiritual practices, social support activities, sleep, and work stress (see Table 2 for items by domain); areas known to mediate the relationships between stress reactivity and physical and mental health. The ALI also includes demographic variables, including hours worked, alcohol use, and health status questions, having a chronic medical condition, taking medication for a chronic medical condition, body mass index (BMI), taking prescription medication for stress or anxiety, days with pain in the past week, and days engaged in hobbies in the past week.

Site leaders completed a survey of onsite physician wellness activities in 2014 and 2016. Physician wellness activities included retreats, nutrition (healthy food options), empathy skills training, conflict resolution/communication skills training, stress management, physical activity options (e.g., exercise rooms), self-regulation skills training (e.g., mind-body skills training), and burnout prevention.

### 2.2. Data Collection and Statistical Analysis

Data were collected directly from the residents online using an individualized link to an internet-based survey website (Survey Monkey, San Mateo, CA, USA) in the first trimester of the first year of residency. Prior to accessing the assessments, residents voluntarily completed an online informed consent form. Statistical analyses were conducted using SPSS v. 24.0 (IBM Corp. Released 2016. IBM SPSS Statistics for Windows, Version 24.0. Armonk, NY, USA).

Descriptive statistics are presented for the wellbeing measures and lifestyle behaviors. A burnout risk group variable was created utilizing medicine norms [15] as follows: high burnout consists of individuals scoring in the high range on both emotional exhaustion and depersonalization; low burnout group includes individuals scoring in the low range on both scales; remaining individuals were categorized as moderate burnout. One-way analysis of variance (ANOVA) with post hoc Tukey tests were conducted to compare wellbeing measures and lifestyle behaviors among burnout groups.

A series of multiple regression analyses were performed to examine the relationship between the lifestyle behavior domains and each of the wellbeing measures. For the lifestyle behaviors, due to variation in response format for some items, scale total scores were calculated utilizing two procedures. For scales where all items were rated on a 0–7 scale (number of days of carrying out the specific lifestyle behavior in the past seven days) the scale scores were formed using mean scores of the items in the same domain. For items where there was variation in response format, such as exercise, social support activities, and alcohol use, items were standardized first and then the means of the *z*-scores were used as the scale scores. Items were reverse-scored where indicated. With the exception of the work stress scale, higher scores indicate engaging in more of the behaviors in that domain. Higher scores on the work stress scale indicate a greater level of work stress. The correlation between the wellbeing measures and gender and marital status were examined. If the correlation was statistically significant, gender and/or marital status was included in the model. The initial regression models included all lifestyle variables and demographic variables, as indicated. A final regression analysis was performed dropping non-significant predictors (*p* > 0.05).

## 3. Results

### 3.1. Sample

The sample consisted of 203 first-year residents from four incoming classes (2012, *n* = 15; 2013, *n* = 88; 2014, *n* = 59; 2015, *n* = 41) at five pediatric residency programs participating in the PIMR program: (1) University of Arizona (*n* = 41); (2) University of Chicago (*n* = 56); (3) Eastern Virginia Medical School/Children’s Hospital of the King’s Daughters (*n* = 55); (4) University of Kansas Medical Center (*n* = 26); and (5) Stanford University (*n* = 25). The survey response rate across the cohorts was 63% and ranged between 45–88% across the residency programs. The sample was predominantly female (76%), white (71%) or Asian (13%), and non-Hispanic (94%) with an average age of 28 years old (range 24–39 years old). Half of the sample was married or cohabitating (50%) and 12% had children. The majority of residents were US Doctors of Medicine (76%), followed by foreign medical graduates (13%) and US Doctors of Osteopathic Medicine (11%). Residents completed the online surveys at the beginning of PGY1. In terms of health status, 25% (*n* = 50) reported a chronic medical condition and 19% (*n* = 38) reported taking medication for a chronic medical condition. The most common medical conditions reported were psychological (depression, anxiety, and/or attention deficit hyperactivity disorder; *n* = 15), asthma (*n* = 12) and allergies (*n* = 9). More than two-thirds (68.2%; *n* = 135/198) had a normal BMI, while 23% (*n* = 46) fell in the overweight range and 5% were in the obese range (*n* = 10). Residents reported experiencing pain an average of 1.3 days in the past seven days (range 0–7). Almost half of the residents (47%; *n* = 95) reported experiencing at least one day of pain. Few residents reported taking prescription medication for stress or anxiety (*n* = 16; 8%). Alcohol consumption averaged 2.8 drinks in the past 7 days, with a maximum of 15 drinks in the past week. Three residents reported smoking cigarettes (2%). In terms of hours worked in the past week, 92% (*n* = 186) reported working 80 h or less with 59% (*n* = 119) working 61–80 h.

### 3.2. Wellbeing Measures

Descriptive statistics for the wellbeing measures are presented in Table 2. The average perceived stress score (16) for this resident sample was slightly higher than normed sample data for the general public (11.9–14.7), but was consistent with ranges for healthcare students (15.5–16.7) [23]. The average depression score (12.9) was within the non-depressed range, with 70% of the sample scoring in this range. However, 30% scored over 16, the cut-off score indicating a risk for clinical depression [14,24]. Over half the sample (55%) scored in the low emotional exhaustion range. Less than half (42%) scored in the low depersonalization range. Twenty percent of residents scored in the high emotional exhaustion range and 32% scored in the high depersonalization range. Fifteen percent scored in the high burnout range on both the emotional exhaustion and depersonalization scales. The average score on personal accomplishment (29.5) was in the low range (high risk for burnout) [15]. The average life satisfaction score was 26.4 (in the satisfied range) with 68.9% scoring in the satisfied to extremely satisfied range [17,18]. The mean positive affect (35.2) and negative affect scores (20.6) were slightly better than the mid-point (30) on the Positive and Negative Affect Schedule (PANAS) scale and consistent with what was observed in incoming family medicine residents in the Integrative Medicine in Residency (IMR) program [25].

The average mindfulness score (35.4) was slightly higher than a general sample [20]. Residents in the current sample scored lower on perspective taking and empathic concern and higher on personal distress than a study of incoming internal medicine residents [26]. The average empathy score in this resident sample (110.75) is lower than means obtained in the Jefferson Empathy Scale (JES) validation study with both resident and medical student samples (118) [22].

### 3.3. Lifestyle Behaviors

Descriptive statistics for the lifestyle behavior items by domain are presented in Table 3. Eating breakfast was the most frequent behavior (mean = 5.7 days), while eating 5 servings of fruits and vegetables was the least frequent (mean = 3.3 days). Most did not drink sugary beverages (60%). Residents reported an average of 2 days of at least 10 min moderate exercise and 1.6 days of 10 min of vigorous exercise. More than half of residents reported either 1–2 days (42%) or 3–4 days (28%) of 30 min of moderate exercise. Most residents (83%) reported sedentary behavior less than 70% on an average day. Residents reported engaging in an activity to manage stress an average of 3.3 days. The most frequent practice was prayer (mean 2.3 days). The least frequent practice was progressive muscle relaxation (mean = 0.2 days). While residents reported spending time in nurturing relationships with family or friends most days (mean = 4.8 days), much less time was spent socializing with friends (mean = 2.1 days). Most residents (74%) reported feeling a sense of belonging to a group. While getting 7–9 h of sleep (mean = 3.4 days) or waking feeling rested (mean = 3 days) averages were somewhat low, trouble staying asleep was not a frequent issue (mean = 1.4 days). Residents reported enjoying work an average of 4.6 days and feeling overwhelmed 2.6 days per week.

### 3.4. Differences between Burnout Risk Groups on Wellbeing and Lifestyle Behaviors

In the analyses examining the impact of burnout risk level on wellbeing, all of the models were statistically significant (*p* < 0.006; Table 4). In comparing the burnout risk groups, the low burnout risk group experienced significantly greater wellbeing than the high-risk group (*p* < 0.05) in all of the models. Specifically, the low burnout risk group experienced less perceived stress, depression, negative affect, and personal distress and greater satisfaction with life, positive affect, mindfulness, empathic concern, perspective taking, and empathy than the high-risk burnout group (*p* < 0.05). Differences between the moderate burnout risk group and the high burnout risk group were also observed for perceived stress, depression, negative affect, satisfaction with life, positive affect, mindfulness, perspective taking and empathy (*p* < 0.05), with the moderate risk group experiencing greater wellbeing than the high-risk group. The low burnout risk group was significantly different from the moderate burnout risk group, experiencing less perceived stress, depression, and negative affect and greater positive affect, empathic concern, and empathy (*p* < 0.05). All post hoc group comparisons were statistically significant in the perceived stress, depression, negative affect, positive affect, and empathy analyses, indicating that wellbeing decreased significantly as burnout risk increased.

For the lifestyle behaviors, the diet/nutrition, social relationships, sleep and work stress models were statistically significant (*p* < 0.05; Table 4). The post hoc group comparisons between the low-risk and high-risk groups or moderate-risk and high-risk groups, were statistically significant (*p* < 0.05) in these models. The low-risk and moderate-risk groups reported engaging in a greater frequency of social behaviors and quality sleep than the high-risk group. For diet/nutrition, the high-risk group reported a lower frequency of healthy eating behaviors than the moderate risk group. In the work stress model, the post hoc comparisons were significantly different between all burnout groups, indicating an increase in work stress as burnout risk increased (*p* < 0.05). There was no difference between the burnout risk groups for exercise, mind-body/spiritual practices, hobbies or alcohol use.

### 3.5. Relationship between Lifestyle Behaviors and Wellbeing Measures

Gender was significantly correlated with empathic concern and empathy and, therefore, included in those regression models. Marital status was correlated with life satisfaction and included in that regression model. The various combinations of lifestyle behaviors contributed 6% to 49% of the variance in the tested models (***R*^2^**), depending on the wellbeing measure examined (see Table 5 and Table 6). Work stress was a statistically significant predictor in all but one regression model, empathic concern. Greater work stress was associated with increased perceived stress, depression, emotional exhaustion, depersonalization, negative affect, personal distress, and lower personal accomplishment, life satisfaction, positive affect, mindfulness, perspective taking, and empathy. The second-strongest predictor across the wellbeing models was exercise. A higher frequency of engaging in exercise was associated with less perceived stress, depression, negative affect, and personal distress, and higher levels of personal accomplishment, positive affect, mindfulness, perspective taking, and empathy. A higher frequency of engaging in social support activities was associated with higher life satisfaction, mindfulness, and empathic concern and less depression. More quality sleep was associated with less perceived stress, depression, emotional exhaustion, and negative affect. A greater frequency of healthy eating behaviors was associated with lower levels of depersonalization and higher life satisfaction. While a higher frequency of engaging in hobbies was associated with decreased personal distress, it was also associated with less mindfulness. Mind-body/spiritual practices were associated with increased mindfulness only. Alcohol use was non-significant in all the models. Gender was associated with empathic concern and empathy, with females having greater empathic concern and empathy, while marital status was non-significant.

### 3.6. On-Site Physician Wellness Activities

**Figure 1:**
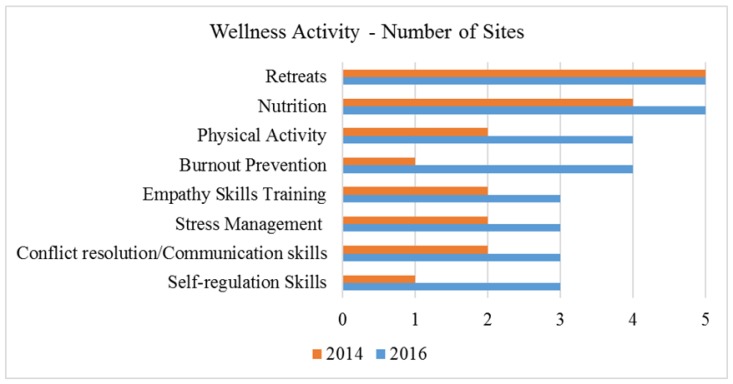
On-site physician wellness activities—number of sites by year.

Wellness retreats were the most frequent type of wellness activity offered, with all sites hosting wellness-focused retreats over the three-year period (see Figure 1). These retreats varied in content-based on-site faculty preferences and available resources. Most were held off-site in a private setting and provided opportunity for small group discussions and peer support, informational seminars, and experiential learning. The next-most frequently reported activity was increased nutrition options for residents, available initially at four sites, and at all sites by 2016. Physical activity options (mainly access to gyms) increased from two sites in 2014 to four sites by 2016. Burnout prevention activities increased from one site in 2014 to four sites; and by 2016, other wellness activities (empathy skills training, self-regulation skills, conflict resolution/communication, and stress management) were offered at three of the five sites.

## 4. Discussion

It is well established that burnout is prevalent in medical trainees and takes a steep toll on mental and physical health [2]. This highlights the need for reform of the current medical education model, and the paradox of recruiting medical students for both their academic strength and empathic qualities. A combination that may predispose talented trainees to distress during the rigors of conventional medical training. Addressing and preventing burnout from the very earliest stages of medical school training is necessary, because burnout impacts patient care, quality of patient counseling, and prevalence of medical errors [7]. The etiology of burnout is complex, involving a variety of factors at the organizational, institutional, and individual levels, requiring coordinated, systems-level solutions [8]. Surveys show that burnout rates in pediatric trainees mirror national prevalence, emphasizing the urgent need for change at the earliest stages of pediatric training in addition to preventive steps and education during medical school to avoid the recurring pitfall of receiving first-year residents already in serious stages of burnout [4,6].

To highlight this point, our study findings demonstrate that a substantial proportion of first-year pediatric residents in the PIMR pilot study began residency with higher levels of burnout and depression and poorer emotional intelligence and empathy than comparison samples, mirroring national trends [6]. Fifteen percent of residents in the study were high risk (high on both emotional exhaustion and depersonalization), 50% were in the moderate risk (high or moderate in either emotional exhaustion or depersonalization), and 35% were at low risk. In addition, with respect to depression, 30% scored in the clinical depression risk range. This is considerably higher than a study of residents and medical students that found 11% of first year residents were in the clinical depression risk range [27]. 

In the cohort, as burnout risk increases, overall wellbeing, empathy and emotional intelligence decreases. Residents with high burnout risk also reported more days of experiencing work stress, and had lower wellbeing, social support, stress management behaviors, emotional intelligence, and empathy than residents in the low and moderate risk groups. Overall, work stress was the strongest predictor of burnout, wellbeing, and emotional intelligence in our sample, significant in all models except empathic concern. The sense of feeling overwhelmed and lack of work enjoyment appears to permeate all areas of the resident’s life, increasing burnout and decreasing wellbeing, emotional intelligence, mindfulness, and empathy.

Residents in the high-risk burnout group reported lower frequencies of healthy eating, social support activities, and quality sleep than their peers. One in four residents were living with a chronic illness, and 28% of residents entering the PIMR program were either overweight (23%) or obese (5%), highlighting the links between chronic stress and upregulation of inflammatory cytokines, depression, and other related comorbidities [28,29,30].

Exercise was a strong predictor of wellbeing and emotional intelligence, associated with less stress and depression, greater personal accomplishment, affect, mindfulness, emotional intelligence and empathy, although not distinguishing between burnout groups. Inadequate sleep was associated with emotional exhaustion, as well as stress, depression, and negative affect, similar to the burnout risk models. Social support activities were associated with less depression and greater life satisfaction, mindfulness, and empathic concern, although contrary to the burnout models, they were not associated with the burnout measures. Higher quality diet/nutrition behaviors were associated with less depersonalization and greater life satisfaction. Female gender was associated with greater empathic concern and empathy.

In our study, mind-body spiritual practices were not associated with any of the burnout, wellbeing, or emotional intelligence measures. One reason for these findings may be due to the low frequency of engagement in these behaviors in this sample. The narrow range may have limited the ability to detect an association to the burnout, wellbeing, and emotional intelligence variables. Fortunately, some residency programs are beginning to teach these behaviors, and future studies will be able to examine the role of mind-body practices in prevention of burnout.

In summary, a majority of residents in the study were at either moderate or high risk of burnout, not meeting basic recommendations for healthy lifestyle habits, and stood a 1 in 4 chance of being overweight or obese. Our findings are consistent with other surveys that document high levels of burnout in graduating US medical students [31], and mirror studies demonstrating lack of regular physical activity in residents [32,33], and published reports of lower levels of stress and burnout in medical students who exercised regularly [34,35,36]. While the percentage of overweight residents was lower in our sample (23%) when compared to a study of first year residents from multiple specialties (34%) [37], the rate of obese residents in our sample of pediatric residents was higher (5% vs. 0%). In addition to the protective lifestyle behaviors mentioned, the remaining wellness activities (empathy skills training, self-regulation skills, conflict resolution/communication, stress management) were offered at 3 of the 5 sites. Ideally, these would serve to help moderate perceived work stress and help build connections at work, cultivating social resilience, which has been shown to be protective against burnout in medical training [38].

National surveys have shown that organizational and institutional measures to reduce and prevent burnout are effective [39,40], and a prudent financial investment [41]. Despite these findings, and widespread recognition of burnout’s mental and physical toll, few programs offer approaches to promote self-care activities associated with lower burnout prevalence [34,42,43,44].

The PIMR program addresses these gaps in two important ways. First, by embedding a curriculum on protective lifestyle behaviors into residency training, the concept of self-care is acknowledged and normalized. Second, engagement of a critical mass of residents and faculty instructors participating in and teaching these activities drives broader culture change within individual organizations. In our study, this is borne out by the increase in program wellness activities over time. Perhaps most notable is the increase from only 1 site offering burnout prevention activities in 2014 to 4 sites with burnout prevention activities in 2016. Newly revised ACGME competencies mandating specific attention to resident wellbeing will hopefully provide critical leverage to move this initiative forward [9].

Furthermore, promotion of healthy lifestyle behaviors in residents aligns with developing themes in the medical literature suggesting that trainees with healthier lifestyle habits are more likely to counsel patients on healthy lifestyle habits, and to do so more effectively than their less healthy colleagues [45,46,47]. This point has special relevance in pediatric trainees, who have the potential to encourage a lifetime of healthy habits in their young patients.

### Limitations

One of the main limitations of this study is the limited generalizability of the findings. The five participating residencies were not randomly selected but volunteered to participate to pilot a new integrative medicine curriculum. Given that there are nearly 200 pediatric residencies nationwide, it is not possible to determine the representativeness of these 5 sites. In addition, the response rate was 63% across the sites, with variation in the response rate between the sites, also limiting our ability to generalize our findings. Data was collected during the first trimester of residency and does not reflect the full measure of residency stressors that accumulate throughout residency.

A second limitation is our primary emphasis on lifestyle behaviors as potential mediators/moderators in the relationship between stress and burnout and its consequent effects. Individual factors that can mediate the impact of stress, such as cognitive appraisals, coping styles and strategies, self-efficacy expectations, grit, optimism, resiliency, hardiness, and social competence were not assessed. System level factors were also not assessed. These include current rotations, prior experience, and levels of faculty support and burnout and how this influences resident burnout, all areas of active study. The newly developed lifestyle behavior instrument used in this study may not have fully captured the lifestyle behaviors most critical to wellbeing. Further, the lifestyle behaviors were captured retrospectively, thus it was not possible to confirm the accuracy of the self-reported lifestyle behaviors with other methods, such as daily recording of the behavior. Lastly, the study utilized online self-report surveys which may be subject to recall bias and social desirability influences.

Future directions could address efforts to increase the reach of the PIMR program by increasing the number of enrolled sites and residents, thereby increasing reach and diversity. Another strategy might include a more tailored rollout of self-care curriculum modules designed to educate leaders and encourage a culture of wellness and engagement within the top levels of organizations [48].

## 5. Conclusions

Accruing research suggests that first-year residents enter training with high levels of burnout, emphasizing the need for effective solutions to address burnout in medical education. Burnout prevalence in our study supported these findings. Furthermore, high burnout risk was associated with a decrease in overall wellbeing, increased work stress, inadequate sleep, fewer social support activities, and poorer diet quality. New mandates from the ACGME to promote resident wellbeing are encouraging, yet few educational programs currently exist to meet these requirements. National surveys show that organizational measures designed to reduce burnout have proven effective. The PIMR program offers an innovative curriculum that residency programs can use to target protective lifestyle behaviors correlated with decreased burnout measures. These include physical activity, sleep, nutrition, and stress management/coping skills. Within the PIMR program, education about these behaviors is delivered in a train the trainer educational model that will ideally equip residents to become more effective role models and counselors to their young patients.

## Figures and Tables

**Table 1:** Wellbeing measures.

Dimension	Measure	Description
Perceived stress	Perceived Stress Scale (PSS) [13]	10 items; scores 0–40
Depression symptoms	Center for Epidemiologic Studies—Depression Scale (CES-D) [14]	20 items; scores range from 0–60; score of 16 or higher indicates clinical syndrome
Burnout	Maslach Burnout Inventory (MBI) [15,16]	22 items; 3 subscales emotional exhaustion (EE), depersonalization (DEP), personal accomplishment (PA)
Life satisfaction	Satisfaction with Life Scale (SWLS) [17,18]	5 items; higher total scores indicate greater life satisfaction
Affect	Positive and Negative Affect Scale (PANAS) [19]	20 items; 2 subscales positive affect (POS), negative affect (NEG); higher score more positive, more negative affect
Mindfulness	Freiberg Mindfulness Inventory (FMI) [20]	14 items, higher score more mindful
Emotional Intelligence	Interpersonal Reactivity Index (IRI) [21]	21 items; 3 subscales perspective taking (PT), empathic concern (EC), personal distress (PD); higher scores greater perspective taking, empathic concern, personal distress
Empathy ^a^	Jefferson Empathy Scale (JES) [22]	20 items, higher score greater empathy
Lifestyle Behaviors	Arizona Lifestyle Inventory [12]	35 items measuring frequency of diet/nutrition, exercise, mind-body/spiritual practices, social support activities, sleep, hobbies, alcohol consumption behaviors

^a^ JES not collected in 2012 class.

**Table 2:** Measures of wellbeing—descriptive statistics.

Wellbeing Measures	*N*	*n*	Mean (SD)/% Yes	Range	Norm Data
Perceived stress	190		16.0 (5.9)	2–31	11.9–14.7 ^1^
CES-D depression	190		12.9 (9.2)	0–42	
Non-depressed		132	69.5%		<16 non-depressed
Clinical depression risk		58	30.5%		≥16
MBI—Emotional exhaustion	203		18.1 (9.0)	0–54	19–26 average
Low emotional exhaustion		112	55.2%		
Moderate emotional exhaustion		50	24.6%		
High emotional exhaustion		41	20.2%		
MBI—Depersonalization	203		7.4 (4.9)	0–30	6–9 average
Low depersonalization		85	41.9%		
Moderate depersonalization		54	26.6%		
High depersonalization		64	31.5%		
MBI—Burnout risk group					
Low risk		70	34.5%		
Moderate risk		102	50.2%		
High risk		31	15.3%		
MBI—Personal accomplishment	203		29.5 (6.3)	12–48	39–34 average
Satisfaction with life	203		26.4 (5.8)	5–35	26–30 satisfied
PANAS Positive	174		35.2 (6.4)	10–50	35.3 ^2^
PANAS Negative	174		20.6 (6.0)	10–42	19.6 ^3^
Mindfulness	191		35.4 (7.1)	16–54	34.52 ^3^, 31.17 ^4^
IRI Empathic concern	190		21.5 (4.0)	11–28	22.2 ^5^
IRI Personal distress	190		10.4 (4.9)	0–25	8.9 ^5^
IRI Perspective taking	189		18.3 (4.3)	2–28	20.6 ^5^
Jefferson empathy	174		110.7 (14.2)	79–140	118 ^6^

^1^ General public validation sample; ^2^ Post Graduate Year (PGY1) Family medicine residents; ^3^ General sample; ^4^ Clinical sample; ^5^ PGY1 Internal medicine residents; ^6^ Resident and medical student mean, SD: standard deviation.

**Table 3:** Lifestyle behaviors—descriptive statistics.

Domain/Items	*N*	*n*	Mean/%	SD	Range
Diet/Nutrition					
5 Servings fruits & vegetables	203		3.3	2.2	0–7
Eat calcium rich foods	203		4.9	2.0	0–7
Eat breakfast	200		5.7	1.9	0–7
Eat home cooked dinner	203		4.2	2.0	0–7
Drank caffeinated beverages *	203		5.6	2.2	0–7
Sugary fluid drinks average day *	202		0.6	0.9	0–5
0		122	60.4%		
1		61	30.2%		
2		8	3.9%		
3		9	4.5%		
4		1	0.5%		
5 or more		1	0.5%		
Servings high fiber average day	203		2.1	1.3	0–5
0		15	7.4%		
1		51	25.1%		
2		67	33.0%		
3		43	21.2%		
4		14	6.9%		
5 or more		13	6.4%		
Vegetarian	188		0.07	0.25	0–1
Yes		13	6.9%		
Exercise					
Vigorous physical activity ≥ 10 min	202		1.6	1.8	0–7
Moderate physical activity ≥ 10 min	202		2.0	2.0	0–7
Moderate physical activity ≥ 30 min	187				
None		28	15.0%		
1–2 days		78	41.7%		
3–4 days		53	28.3%		
5–6 days		21	11.2%		
Everyday		7	3.7%		
Percent sedentary average day *	198				
Less than 5%		4	2.0%		
6–10%		8	4.0%		
11–20%		12	6.1%		
21–30%		19	9.6%		
31–40%		25	12.6%		
41–50%		36	18.2%		
51–60%		29	14.6%		
61–70%		31	15.7%		
71–80%		25	12.6%		
81–90%		9	4.5%		
91–100%		0	0%		
Mind-Body/Spiritual Practices					
Activity to relax or manage stress	202		3.3	2.4	0–7
Prayer	202		2.3	2.8	0–7
Spiritual ritual non-prayer	202		0.6	1.7	0–7
Personal reflection	201		1.5	2.2	0–7
Breathing for stress reduction	202		0.5	1.3	0–7
Progressive muscle relaxation	191		0.2	0.8	0–7
Social Support Activities					
Spend time family/friends	203		4.8	2.3	0–7
Receive healthy touch	203		4.4	2.6	0–7
Socialize with friends	202		2.1	1.6	0–7
Sense of belonging groups	203				
Yes		150	73.9%		
Not sure		20	9.9%		
No		33	16.3%		
Number groups belong	203		1.9	1.2	0–6
*Sleep*					
Get 7–9 h of sleep	203		3.4	2.2	0–7
Wake feeling rested	201		3.0	2.1	0–7
Trouble staying asleep *	201		1.4	2.0	0–7
Hobbies	202		2.1	2.3	0–7
Number of Alcohol Drinks	200		2.8	3.0	0–15
Work					
Enjoy work *	203		4.6	1.9	0–7
Feel overwhelmed by work	203		2.6	2.2	0–7

* Items reverse-scored when creating scales.

**Table 4:** Results of one-way variance analysis (ANOVA) for Burnout Group and wellbeing—means and standard deviations.

Wellbeing Measures	Total *N* *	Low Risk *n* = 70		Moderate Risk *n* = 102		High Risk *n* = 31		*p*-Value
		Mean	SD	Mean	SD	Mean	SD	
Perceived Stress	190	12.6 ^a,c^	5.1	16.4 ^b^	4.9	22.7	4.4	<0.001
CES-D Total	190	8.1 ^a,c^	5.7	12.6 ^b^	8.0	25.2	8.3	<0.001
Satisfaction with Life	203	27.9 ^a^	4.3	27.0 ^b^	5.3	21.1	7.4	<0.001
PANAS Positive	174	38.0 ^a,c^	4.5	35.4 ^b^	6.4	28.6	4.6	<0.001
PANAS Negative	174	17.5 ^a,c^	4.6	20.5 ^b^	5.2	27.8	4.9	<0.001
FMI Mindfulness	191	37.6 ^a^	6.9	35.1 ^b^	7.1	31.6	6.4	0.001
IRI Empathic Concern	190	22.8 ^a,c^	3.7	21.2	3.9	19.7	3.9	0.001
IRI Perspective Taking	189	19.5 ^a^	4.1	18.2 ^b^	3.8	15.5	5.2	<0.001
IRI Personal Distress	190	9.3 ^a^	4.7	10.4	4.8	12.7	4.7	0.006
Jefferson Empathy	174	115.8 ^a,c^	13.0	110.6 ^b^	13.2	99.2	14.1	<0.001
**Lifestyle Behaviors**								
Diet/Nutrition	203	0.084	0.51	0.043 ^b^	0.40	−0.20	0.62	0.048
Exercise ^d^	203	0.045	0.7	0.053	0.7	−0.25	0.6	0.095
Mind-body/Spiritual Practices	203	1.6	1.2	1.4	1.0	1.1	1.1	0.13
Social Support Activities ^d^	203	0.018 ^a^	0.7	0.075 ^b^	0.6	−0.30	0.7	0.012
Sleep	203	4.4 ^a^	1.5	4.1 ^b^	1.4	2.9	1.3	<0.001
Hobbies	202	2.3	2.6	2.0	2.1	1.8	2.1	0.56
Alcohol drinks	200	2.5	2.7	3.2	2.9	2.5	3.6	0.32
Work Stress	203	1.7 ^a,c^	1.1	2.6 ^b^	1.5	4.2	1.6	<0.001

* *N* varied by measure. Post hoc Tukey tests: ^a^ low-risk vs. high-risk group, *p* < 0.05; ^b^ moderate-risk vs. high-risk group, *p* < 0.05; ^c^ low-risk vs. moderate-risk group, *p* < 0.05; ^d^ means for exercise and social relationships are *z*-scores, therefore the group mean is zero. Means greater than 0 indicate higher frequency than the group mean, while negative means indicate a frequency lower than the group mean.

**Table 5:** Lifestyle behavior predictors, *R*^2^ and betas on measures of wellbeing ^a^.

Wellbeing Measure	Model Adjusted *R*^2^	Model *p*-Value	*β*	*t*	*p*-Value
Perceived stress	0.39	<0.001			
Work stress			0.50	7.95	<0.001
Exercise			−0.19	−3.35	0.001
Sleep			−0.16	−2.45	0.015
CES-D Total	0.49	<0.001			
Work			0.47	7.80	<0.001
Sleep			−0.23	−3.93	<0.001
Social			−0.18	−3.14	0.002
Exercise			−0.13	−2.38	0.018
MBI emotional exhaustion	0.40	<0.001			
Work stress			0.56	9.52	<0.001
Sleep			−0.17	−2.94	0.004
MBI Depersonalization	0.16	<0.001			
Work stress			0.34	5.21	<0.001
Diet			−0.20	−3.09	0.002
MBI personal accomplishment	0.18	<0.001			
Work Stress			−0.34	−5.32	<0.001
Exercise			0.23	3.64	<0.001
Satisfaction with life ^b^	0.26	<0.001			
Work stress			−0.36	−5.65	<0.001
Social			0.21	3.29	0.001
Diet			0.16	2.60	0.010
PANAS positive	0.31	<0.001			
Work stress			−0.46	−7.29	<0.001
Exercise			0.27	4.25	<0.001
PANAS negative	0.26	<0.001			
Work stress			0.37	5.05	<0.001
Exercise			−0.17	−2.64	0.009
Sleep			−0.17	−2.34	0.021
Mindfulness	0.20	<0.001			
Work stress			−0.27	−3.95	<0.001
Exercise			0.25	3.66	<0.001
Social			0.15	2.18	0.031
Hobbies			−0.16	−2.27	0.024
Mind-body			0.14	2.01	0.046
IRI empathic concern ^c^	0.06	0.001			
Gender			0.20	2.77	0.006
Social			0.17	2.41	0.017
IRI perspective taking	0.064	0.001			
Work stress			−0.20	−2.76	0.006
Exercise			0.18	2.47	0.014
IRI Personal distress	0.078	<0.001			
Exercise			−0.21	−2.94	0.004
Work stress			0.20	2.78	0.006
Hobbies			−0.15	−1.97	0.050
Jefferson empathy ^c^	0.17	<0.001			
Gender			0.29	4.11	<0.001
Exercise			0.26	3.72	<0.001
Work stress			−0.17	−2.47	0.009

^a^ Only statistically significant (*p* < 0.05) predictors in final model are presented. b Due to the correlation with marital status, marital status was included in the initial model. However, it was non-significant and was dropped from the final model. Married/cohabitating is coded as 1, single as 0. ^c^ Due to the correlation with gender, gender was included in the model. Gender is coded 1 for male, 2 for female.

**Table 6:** Lifestyle behavior predictors and relationship to wellbeing measures.

Lifestyle Behavior	PSS	CES-D	MBI-EE	MBI-DEP	MBI-PA	SWLS	PANAS Positive	PANAS Negative	FMI	IRI EC	IRI PT	IRI PD	JES
Work Stress	↑	↑	↑	↑	↓	↓	↓	↑	↓		↓	↑	↓
Exercise	↓	↓			↑		↑	↓	↑		↑	↓	↑
Social Support		↓				↑			↑	↑			
Sleep	↓	↓	↓					↓					
Diet/Nutrition				↓		↑							
Hobbies									↓			↓	
Gender										↑			↑
Mind-body/Spiritual									↓				
Alcohol drinks													

↑ Positive relationship between wellness behavior and wellbeing measure; ↓ Negative relationship between wellness behavior and wellbeing measure.
